# Pulmonary sarcomatoid carcinoma mimicking pleural mesothelioma: A case report

**DOI:** 10.1097/MD.0000000000027813

**Published:** 2021-11-12

**Authors:** Le Wang, Jing Zhang, Xing Chen, Maoli Liang, Shuo Li, Wei Zhou, Jie Cao

**Affiliations:** Department of Respiratory and Critical Care Medicine, Tianjin Medical University General Hospital, Tianjin, China.

**Keywords:** biopsy, histopathology, immunohistochemistry, pleural mesothelioma, pulmonary sarcomatoid carcinoma

## Abstract

**Introduction:**

Pulmonary sarcomatoid carcinoma (PSC) is an extremely rare biphasic tumor characterized by a mixture of malignant epithelial and mesenchymal cells. Owing to the rarity, as well as the lack of typical manifestations and imaging signs, the rate of misdiagnosis is high. Herein, we present a case of PSC misdiagnosed as pleural mesothelioma in a 59-year-old man.

**Patient concerns:**

A 59-year-old man presented with recurrent coughing, fever, and chest pain.

**Diagnosis:**

Chest computed tomography showed 2 large and dense masses involving the inferior lobes of right lung, along with slight irregular pleural thickening and a small amount of effusion.

**Interventions:**

Chest computed tomography-guided tumor biopsy was performed. PSC was confirmed based on histopathology and immunohistochemistry. The patient refused treatment due to economic reasons.

**Outcomes:**

The patient developed adrenal, multiple lung and brain metastasis. The overall survival time was 11 months.

**Conclusions:**

PSC, despite its rarity, should be considered in the differential diagnosis of lung cancer. Besides, biopsy, histopathology, and specific immunohistochemical staining of larger tissue specimens can be contributing to the accurate diagnosis of PSC.

## 1 Introduction

Pulmonary sarcomatoid carcinoma (PSC) is a monoclonal-origin epithelial tumor with stronger invasion, higher malignancy and poorer differentiation, accounting for 0.4% of all lung tumors.^[1]^ Owing to the rarity, as well as the lack of typical manifestations and imaging signs, the rate of misdiagnosis is high. In particular, it still remain an important diagnostic challenge for clinical physicians to differ PSC from pleural mesothelioma (PM). PSC is one of poorly differentiated nonsmall-cell lung carcinomas (NSCLCs) that contain at least 10% sarcoma or sarcoma-like (spindle and/or giant cell) differentiation. PM is one of poorly differentiated malignant tumor arising from mesothelial cells (MCs), and also showing spindle cells or mesenchymal morphology. Both tumors often show high-grade histology without specific morphologic features.^[2]^ PSC grows more aggressively in pulmonary parenchyma than other NSCLCs, often involving the pleura and the chest wall, making it difficult to distinguish from PM growing along the parietal and visceral pleura.^[3,4]^ It was also reported that some PSC cases even showed prominent chest wall and pleural tumors with obscure primary lung tumors.^[5]^ Especially, in the PSC cases that epithelial components cannot be detected from the tissue obtained by needle biopsy, the differential diagnosis between and PSC and PM was more difficult.^[3]^ Herein, we report a PSC case, initially misdiagnosed as PM, was finally confirmed by histopathology and specific immunohistochemical staining examination.

## 2 Case report

On July 30, 2013, a 59-year-old man was admitted to our department for fever, coughing, dyspnea, and consistent right chest pain for 20 days. He had a 40-year history of cigarette smoking (25 cigarettes per day) and medical history of hypertension and coronary heart disease.

**Figure 1:**
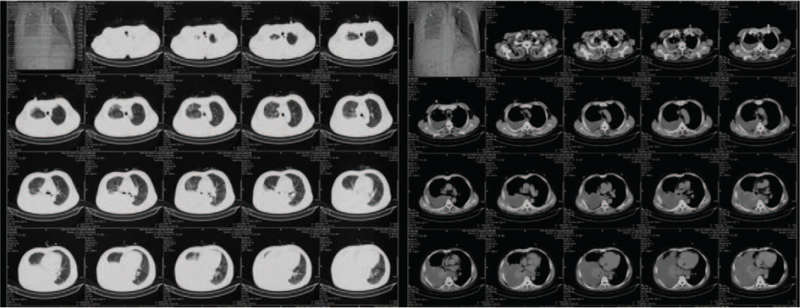
A large, dense mass involving the middle and inferior lobes of right lung can be observed, along with pleural thickening and pleural effusion in the nonenhanced CT imaging of chest. CT = computed tomography.

Chest computed tomography (CT) performed at another hospital before admission to our department revealed a large, dense mass involving the middle and inferior lobes of right lung, along with pleural thickening and effusion (Fig. 1). Thoracentesis and pleural biopsy were performed simultaneously. Inflammatory cellulosic exudate, MC proliferation, and a few heteromorphic cells were observed in pleural fluid sediment. Immunohistochemistry showed positive expression of MC and P53, and negative expression of carcino embryonic antigen (CEA) and thyroid transcription factor-1 (TTF-1). The pleural biopsy specimen comprised striated muscle, hyperplastic fibrous connective tissue, and epithelial cells. Immunohistochemistry showed positivity for keratin and vimentin, and negativity for CEA, MC, P53, TTF-1, Ki-67, and calretinin, this indicating the possibility of mesothelioma. The patient was referred to our department for further diagnosis and treatment.

**Figure 2:**
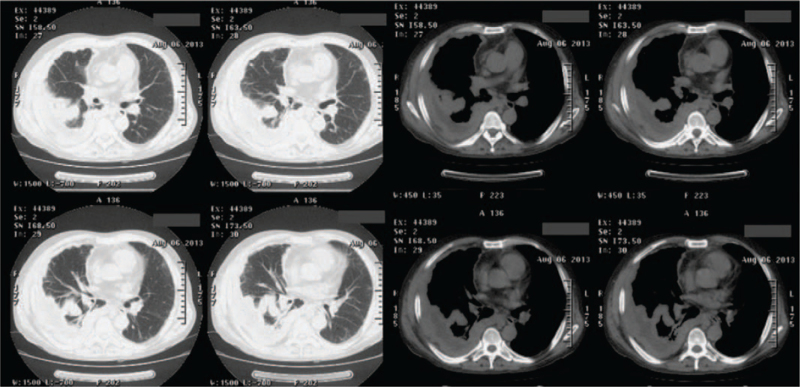
Irregular pleural thickening, little hydrothorax, and a large mass occupying the middle and inferior lobe of right lung are visible in the nonenhanced CT imaging of chest. CT = computed tomography.

On physical examination in our department, the patient's body temperature was 38.2°C. Breath sounds were low in the right lobe, while the left lobe was filled with wheezing. Chest CT revealed irregular pleural thickening, little hydrothorax in the right pleural cavity, and a large mass occupying the middle and inferior lobes of right lung (Fig. 2). The patient's symptoms resolved slightly after treatment with antibiotics and antitussive and expectorant drugs. After that, he refused further examination and was discharged.

**Figure 3:**
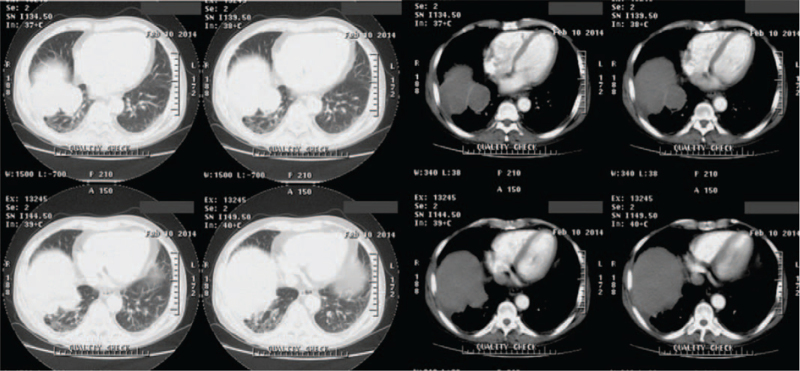
Two, large, well-defined masses occupying the inferior lobe of right lung, along with slight pleural thickening and little pleural effusion are showed by the enhanced chest CT. CT = computed tomography.

**Figure 4:**
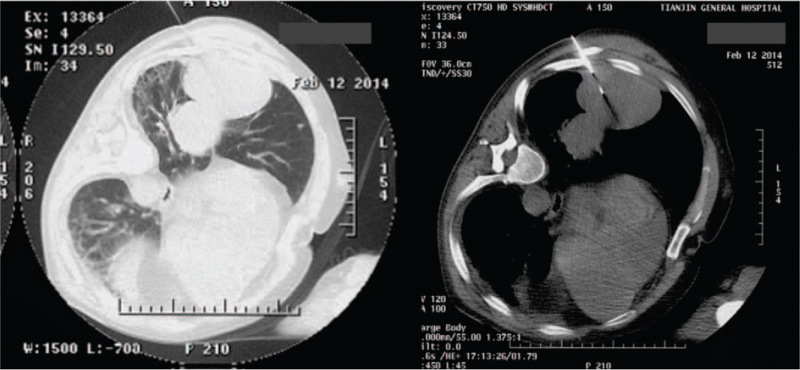
Lung lesion biopsy was performed under chest CT guidance. CT = computed tomography.

**Figure 5:**
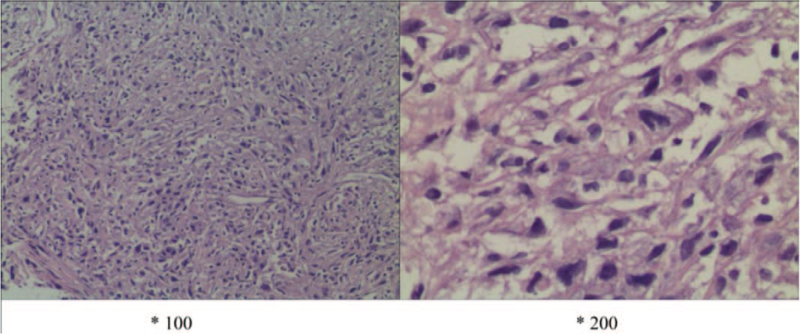
Microscopic findings of lung biopsy specimens. Routine histology, with hematoxylin–eosin staining, showed malignant spindle cells (sarcoma-like features), with nuclear polymorphism and mitotic figures (×100/200).

The patient was followed up regularly every month after discharge. The general condition was passable, with occasional dry coughing, no fever and wheezing. Until February 4, 2014, the patient was again admitted to our department due to fever and aggravation of coughing. Chest enhanced CT revealed 2 large, well-defined masses occupying the right inferior lobe, along with slight pleural thickening and little pleural effusion (Fig. 3). For definitive diagnosis, CT-guided tumor biopsy was performed on February 12, 2014 (Fig. 4). Microscopic examination of the surgical specimen showed malignant spindle cells (sarcoma-like features), with nuclear polymorphism and mitotic figures (Fig. 5). Immunohistochemistry revealed that the lesion was positive for cytokeratin (CK), CK7, and vimentin; focally positive for P63; and negative for epithelial membrane antigen (EMA), smooth muscle actin, TTF-1, desmin, calretinin, and D2-40. Finally, the patient was diagnosed with PSC. Considering the expected poor prognosis and high treatment costs, the patient declined further treatment.

Right adrenal metastasis was found at the 1-month follow-up, and multiple lung metastases at the 3-month follow-up. At 4 months since discharge, the patient had seizures that resulted in unconsciousness, and he died. The total survival was 11 months since presentation of the first symptoms.

## 3 Discussion

In the case, PSC, misdiagnosed as PM at first, was finally confirmed by histopathology and immunochesmistry of larger specimens, highlighting the importance of biopsy and specific immunohistochemical staining of larger tissue specimens at diagnosis.

PSC is one of poorly differentiated NSCLCs containing a component of true sarcoma or sarcoma-like elements.^[6]^ The World Health Organization classified PSC into pleomorphic carcinoma, spindle cell carcinoma, giant cell carcinoma, carcinosarcoma, and pulmonary blastoma.^[7]^ PSC is predominantly in middle-aged and elderly (>65 years old) male smokers with a history of moderate to heavy tobacco consumption.^[8,9]^ In our case, the patient was a 59-year-old man with a 40-year history of cigarette smoking.

There is no specific clinical presentation. Patients may present with chest pain, coughing, hemoptysis, dyspnea, fever, and weight loss, which were similar to that of other respiratory diseases.^[10]^ The patient in our case suffered from recurrent fever, coughing, and chest pain.

CT features of PSC are a mix of sarcoma and carcinoma: large size and smooth edges are features of sarcoma; lobulation, bronchial truncation, and necrosis are features of carcinoma.^[11]^ PSC is more common in the upper lobe of right lung and is usually the peripheral type,^[12]^ which is prone to invading the pleura, thereby manifesting as irregular pleural thickening and pleural effusion, and being misdiagnosed as PM easily. Contrast-enhanced CT features are relatively typical: the peripheral part of the lesion is usually ring-shaped or shows partial enhancement, and the central area usually shows no or slight enhancement and may be necrotic and/or bleeding.^[13]^ In our case, the mass occupied the lower lobe of the right lung, which was initially combined with a small amount of pleural effusion and irregular pleural thickening. Enhanced CT showed slight ring-shaped enhancement in the peripheral part of mass and no obvious enhancement in the central part.

The final diagnosis of PSC depends on the histopathology and specific immunohistochemical staining of surgical or biopsy specimens.^[6]^ It is very difficult to diagnose PSC in small biopsies or cytology,^[6]^ World Health Organization recommend histopathology in combination with immunohistochemistry of sufficient size specimens to facilitate the diagnosis.^[14]^ In our case, the patient only received pleural biopsy and thoracentesis at first, and was misdiagnosed as PM. Therefore, for peripheral PSC invading the pleura, lung biopsy and pleura biopsy should be taken simultaneously to avoid misdiagnosis. It can be seen a mixture of carcinoma and sarcoma/sarcoma-like components in PSC at the microscopic level. The tumors often co-express epithelial markers, such as, CK, EMA, TTF-1, and CEA; as well as, mesothelial markers, such as vimiten and desmin.^[12,15]^ It was reported that immunostaining positive for P63 is also another useful marker for diagnosing PSC.^[16]^ Calretinin, D2-40, TTF-1, and P40 are important immunohistochemical markers to distinguish sarcomatoid PM from PSC. In our case, we found that the tumor specimen was positive for CK, CK7, P63, and vimentin, but negative for EMA, TTF-1, smooth muscle actin, desmin, carletinin, and D2-40. This result suggested that our case had sarcomatoid components and epithelial phenotype, which was in accordance with previous reports.^[10,15,17,18]^

PSC is characterized by faster growth, higher invasion, recurrence, metastasis, and mortality. The median survival period is only 10 to 13.3 months, and the 5-year survival rate varies between 11% and 21%, which is much lower than other types of lung cancers.^[17,19,20]^ However, until now, there has been no effective therapy for PSC. Surgery is still the first choice for the treatment of early PSC, like other NSCLCs. PSC is not sensitive to traditional radiotherapy and chemotherapy, which may be related to the mechanism of epithelial–mesenchymal transition.^[21–23]^ It was reported that the alteration of TP53, EGFR, KRAS, MET, and ALK genes could be indentified in PSC, which provide a molecular basis for targeted therapy.^[22–29]^ In addition, recent investigations have shown high level of PD-L1 expression in PSC, which may provide a rationale for the potential use of immunotherapy.^[18,30–33]^

## 4 Conclusions

PSC is rare, aggressive, poorly differentiated NSCLC, with poor prognosis. Owing to the rarity, as well as the lack of typical manifestation and imaging signs, the rate of misdiagnosis is high. We reported an unusual case of PSC mimicking PM, emphasizing the importance of biopsy, histopathology, and specific immunohistochemical staining of larger tissue specimens at diagnosis.

## Acknowledgments

We would like to thank Editage for English language editing.

## Author contributions

**Conceptualization:** Le Wang, Jing Zhang, Maoli Liang, Shuo Li, Wei Zhou, Jie Cao.

**Data curation:** Le Wang, Jing Zhang, Xing Chen, Maoli Liang, Shuo Li, Wei Zhou.

**Formal analysis:** Le Wang.

**Funding acquisition:** Jie Cao.

**Investigation:** Le Wang, Jing Zhang, Xing Chen.

**Methodology:** Le Wang, Xing Chen.

**Supervision:** Jie Cao.

**Validation:** Maoli Liang, Wei Zhou.

**Visualization:** Shuo Li.

**Writing – original draft:** Le Wang, Jing Zhang.

**Writing – review & editing:** Jie Cao.
